# Effect of Home Bleaching on Microleakage of Fiber-reinforced and Particle-filled Composite Resins

**DOI:** 10.5681/joddd.2013.034

**Published:** 2013-12-18

**Authors:** Farahnaz Sharafeddin, Samira Zare, Zahra Javnmardi

**Affiliations:** ^1^Associate Professor, Biomaterial Research Center, Department of Operative Dentistry, School of Dentistry, Shiraz University of Medical Sciences, Shiraz,Iran; ^2^Asistant Professor, Department of Operative Dentistry, School of Dentistry, Shiraz University of Medical Sciences, Shiraz, Iran; ^3^Postgraduate Student, Department of Orthodontics, School of Dentistry, Shiraz University of Medical Sciences, Shiraz, Iran

**Keywords:** Bleaching, composite resin, fiber-reinforced, filler, microleakage

## Abstract

***Background and aims.*** Bleaching may exert some negative effects on existing composite resin restorations. The aim of this study was to evaluate the effect of home bleaching on microleakage of fiber-reinforced and particle-filled composite resins.

***Materials and methods.*** Ninety class V cavities (1.5×2×3 mm) were prepared on the buccal surfaces of 90 bovine teeth. The teeth were randomly divided into 6 groups (n=15) and restored as follows: Groups 1 and 2 with Z100, groups 3 and 4 with Z250, and groups 5 and 6 with Nulite F composite resins. All the specimens were thermocycled. Groups 1, 3 and 5 were selected as control groups (without bleaching) and the experimental groups 2, 4 and 6 were bleached with 22% carbamide peroxide gel. All the samples were immersed in 2% basic fuchsin dye for 24 hours and then sectioned longitudinally. Dye penetration was evaluated under a stereomicroscope (×25), at both the gingival and incisal margins. Data were analyzed using Kruskal-Wallis, Mann-Whitney and Wilcoxon tests (a=0.05).

***Results.*** Statistical analyses revealed that bleaching gel increased microleakage only at gingival margins with Z250 (P=0.007). Moreover, the control groups showed a statistically significant difference in microleakage at their gingival margins. Nulite F had the maximum microleakage while Z250 showed the minimum (P=0.006).

***Conclusion. ***Microleakage of home-bleached restorations might be related to the type of composite resin used.

## Introduction

Microleakage is the major focus of research studies to improve the durability of composite resin restorations.^1^ Marginal discoloration, recurrent caries, pulpal irritation and tooth hypersensitivity are common problems associated with microleakage.^2^ It has been reported that some changes happen in composite resin restorations following contact with bleaching agents. These restorations may exhibit an increase in superficial roughness and clefts, changes in microhardness and subsequently an increase in marginal microleakage.^3,4^ Bailey and Swift reported that bleaching procedures affect the microparticle composite resins negatively due to their higher concentration of organic matrix compared to hybrid composite resins. They observed cracks between the resin matrix and particles in SEM analysis.^5^

Postoperative bleaching of composite resin restorations with 35% hydrogen peroxide or 10-16% carbamide peroxide gel negatively affected the marginal seal at both enamel and dentin margins; however, other studies have not shown any increase in microleakage, at least not at enamel margins.^6^ Carbamide peroxide is less potent than hydrogen peroxide and exerts less adverse effects on tooth structures.^7^ It is also speculated that since carbamide peroxide breaks down quickly into hydrogen peroxide and urea and urea is primarily responsible for raising PH, it can decrease the adverse effects of bleaching gels.^8^

A current improvement in composite resins is the incorporation of coarse glass fibers, in addition to or instead of conventional inorganic filler particles. The most frequent fiber reinforcements are glass and carbon fiber bundles. Mixing of dental polymers with fibers has the benefit of improved mechanical characteristics and moreover it may act as a crack stopper.^9^

However, clinical failures due to the disruption of the bonded interface are still a common occurrence. Such interfacial defects may arise as a consequence of long-term thermal and mechanical stress or due to stresses generated by composite resin polymerization shrinkage during restorative procedures.^10^ Factors, which can influence polymerization shrinkage include inorganic filler content, molecular weight of the monomer system and its degree of conversion.^11^ Still, it is not clear whether carbamide peroxide affects the marginal seal of restorations and whether replacement of affected restorations is necessary.

The aim of this study was to evaluate the effect of a 22% carbamide peroxide home bleaching gel on microleakage of three composite resin restorations with different matrix compositions: Z100 as a hybrid Bis-GMA-based composite resin, Z250 as a particle-filled Bis-EMA-based composite resin, and Nulite F as a hybrid glass fiber-reinforced and Bis-GMA-based composite resin.^9,12,13^ The null hypothesis was that these three composite resins have similar microleakage with or without bleaching.

## Materials and Methods

Materials used in this study are presented in Table 1. Ninety intact bovine incisors were selected and cleaned of all soft tissue remnants with a surgical blade and a periodontal scaler and then stored in 0.2% thymol solution at 37ºC for one week. Class V cavities (1.5×2×3 mm) were prepared on the buccal surfaces of the teeth (diamond fissure bur 835 010, Dia Swiss, Geneva, Switzerland). The incisal margins were prepared at the enamel and the gingival margins were placed 1 mm below the cementoenamel junction (CEJ). The teeth were randomly divided into 6 groups (n=15) and restored according to Table 2. The cavities were etched with 36% phosphoric acid gel (Dentsply Detrey GmbH, Germany) for 15 seconds, rinsed under tap water for 10 seconds, and gently dried with a mild air jet for 3‒5 seconds (wet bonding technique). In groups 1 to 4 (G1 to G4) two layers of Single Bond (3M ESPE, St. Paul, MN, USA) were applied on the cavity with a microbrush; a mild air jet was blown for 5 seconds to disperse the bonding layer, and then light-cured for 10 seconds using a halogen light-curing unit (Coltulux 50, Coltene/Whaldent Inc, USA) at a light intensity of 450 mW/cm^2^. In G1 and G2, the hybrid composite resin, Z100 (3M ESPE, St. Paul, MN, USA), was placed in the cavity in two increments and each layer was light-cured for 40 seconds. The samples in G3 and G4 were restored in the same manner with the particle-filled composite resin (PFC), Z250 (3M ESPE, St. Paul, MN, USA). In G5 and G6, 2 layers of SP-Bond (Biodental Technologies Pty Ltd, Australia) were applied with a microbrush; each layer was gently air-blown for 3-5 seconds to disperse the bonding layer; and then light-cured for 10 seconds. Similarly, they were restored with the fiber-reinforced composite resin (FRC), Nulite F (Biodental Technologies Pty Ltd, Australia) and then light-cured. The restorations were polished with pop-on Sof-Lex disks (3M ESPE, USA) and subsequently, all the groups were thermocycled for 500 cycles at 5±2/55±2ºC with a dwell time of 30 seconds in each bath. The specimens were then stored in distilled water at 37°C for 7 days.

**Table 1:** Composition of composite resins tested in the study

Composite resin	Shade	Type	Resin Composition	Filler composition and size	Filler volume	Manufacturer
Z100	A2	Hybrid Composite Resin	Bis-GMA, TEGDMA	ZrO2–SiO2 0.01–3.5 μm	66%	3M ESPE, St.Paul, MN, USA
Z250	A2	Particle-filled Composite Resin	Bis-EMA, UDMA, Bis-GMA	ZrO2–SiO2 0.01–3.5 μm	60%	3M ESPE, St.Paul, MN, USA
Nulite F	A2	Fiber-reinforced Composite Resin	Bis-GMA	Glass fiber filler < 9 mm Submicron silica and other glasses	71%	Biodental Technologies Pty Ltd, Australia

**Table 2:** The restorations used in each study groups

Groups	Restoration	Treatment
Group 1	Etch + SingleBond + Z100	No
Group2	Etch + SingleBond + Z100	Bleached
Group3	Etch + SingleBond + Z250	No
Group 4	Etch + SingleBond + Z250	Bleached
Group 5	Etch + SP bond + Nulite F	No
Group 6	Etch + SP bond + Nulite F	Bleached

G2, G4 and G6 were bleached with 22% carbamide peroxide home bleaching gel (WHITEsmile, WHITEsmile GmbH, Germany) for twelve times, 2 hours each time, according to manufacturer’s instructions. In each application, the bleaching gel was injected on fillings and their margins and covered with clear protecting sheets. The teeth were rinsed under tap water and stored in distilled water subsequently.

The apices of the teeth were sealed with light-cured composite resin; the coronal and radicular surfaces of the teeth, except for the restoration and 1 mm around the margins, were covered with two layers of nail varnish and then immersed in 2% basic fuchsin dye at 37°C.

**Figure 1.**
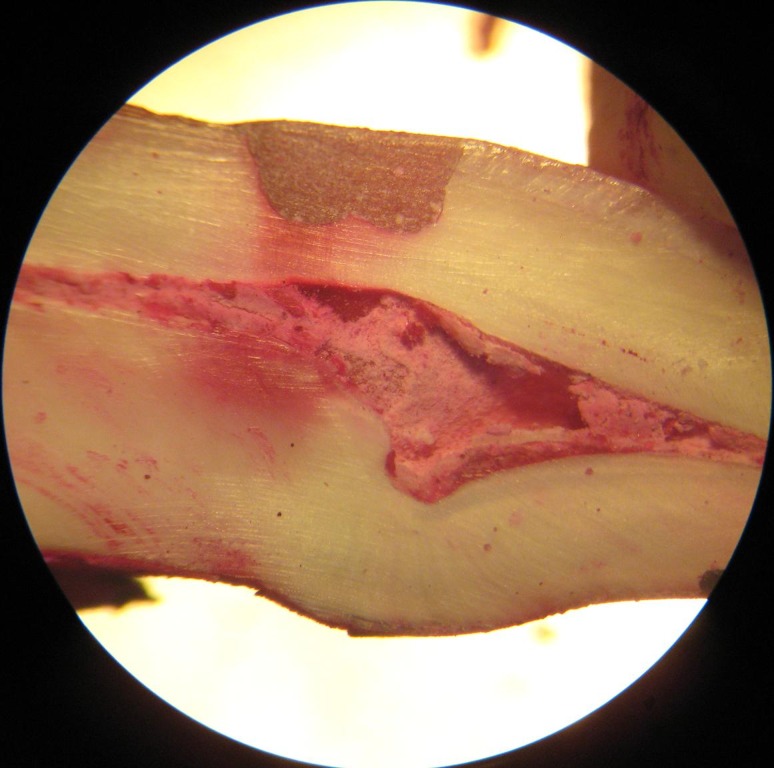
Gingival microleakage, dye penetrated to the axial wall (score 4) and no incisal microleakage (score 0).

After 24 hours, they were washed, dried and sectioned longitudinally in a labiolingual direction at the middle of the restorations with a diamond disk. Dye penetration was evaluated under a stereomicroscope (Motic K-500L, Motic Incorporation Ltd, Hong Kong) at ×25 (Figure 1). Scoring was carried out according to criteria proposed by Soares et al (Table 3).^14^

**Table 3:** Microleakage scoring

0	No dye penetration
1	Dye penetrate up to one third of the cavity depth
2	Dye penetrate one third to two thirds of the cavity depth
3	Dye penetrate more than two thirds of the cavity depth but not reach the axial wall
4	Dye penetrate the axial wall

Data were analyzed by Mann-Whitney *U* test followed by non-parametric Kruskal-Wallis and Wilcoxon tests. Statistical significance was set at P<0.05.

## Results

**Figure 2.**
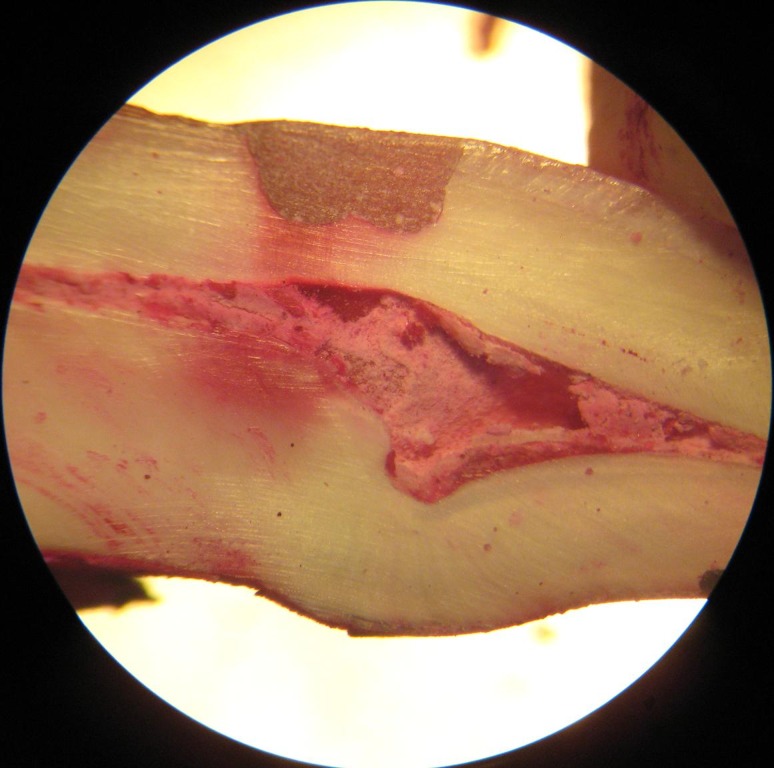
Gingival microleakage, dye penetrated to the axial wall (score 4) and no incisal microleakage (score 0).

Microleakage scores of all the groups are listed in Table 4 and the microleakage medians are presented in Figure 2. Mann-Whitney *U* test showed that gingival microleakage in G4 (bleached Z250) increased significantly after bleaching (P=0.007). Regarding incisal and gingival margins, non-parametric Kruskal-Wallis analysis revealed that there was a statistically significant difference in gingival margins of the control groups (P= 0.006). G5 (unbleached Nulite F) had more and G3 (unbleached Z250) had lower microleakage than G1 (unbleached Z100). According to Wilcoxon test, the microleakage at gingival margins was higher than that at incisal margins (P>0.001) in both bleached and unbleached groups.

**Table 4:** microleakage scores in gingival and incisal margins

Margins	Gingival	Incisal
Scores												
Groups	0	1	2	3	4	Mean (SD)	0	1	2	3	4	Mean (SD)
G1	5	2	1	3	4	1.93 (1.71)^b^	13	0	0	0	2	0.53 (1.4)^c^
G2	5	4	1	2	3	1.6 (1.59)^b^	11	1	2	0	1	0.6 (1.18)^c^
G3	11	4	0	0	0	0.27 (0.45)^a^	15	0	0	0	0	0 (0)^ac^
G4	4	5	0	1	5	1.87 (1.53)^b^	14	1	0	0	0	0.07 (0.26)^c^
G5	5	1	1	1	7	2.27 (1.87)^b^	12	1	0	0	2	0.6 (1.4)^c^
G6	6	4	1	0	4	1.47 (1.68)^b^	14	1	0	0	0	0.07 (0.26)^c^
G1: Z100 control. G2: Z100 bleached. G3: Z250 control. G4: Z250 bleached.
G5: Nulite F control. G6: Nulite F bleached.
The same superscripted letters indicate no significant differences.

## Discussion

Bleaching is a conservative procedure to restore the esthetic appearance of either stained or darkened teeth but it may exert some negative effects on existing composite resin restorations. Although bleaching could change the surface hardness and roughness and color susceptibility of composite resin restorations,^3,4^ it is still not clear whether carbamide peroxide affects their marginal seal and whether replacement of the affected restorations is necessary.

According to the results of the present study, bleached groups G2 and G4 showed more and G6 showed less microleakage at incisal margins compared to their corresponding control groups, with no statistically significant differences. At their gingival margins, G2 and G6 showed less and G4 showed more microleakage compared to their corresponding control groups, with statistically significant differences only in G4 (bleached Z250).

Similar to the present study, Jacob et al reported that postoperative bleaching could increase microleakage in Z250 bonded with Single Bond^15^ while Sartori et al refuted this in their study.^16^ Ayad et al and Mortazavi et al also reported that bleaching could increase microleakage in composite resin restorations,^17,18^ but White et al and Klukowska et al discovered that bleaching did not influence microleakage of Z250, bonded with Scotchbond I.^19,20^ Khoroushi et al also reported that bleaching did not influence microleakage of existing Z100 restorations bonded with Single Bond,^21^ consistent with the results of the present study.

The majority of dental composite resins are composed of a resin matrix, primarily Bis-GMA (bisphenol-A diglycidyl ether dimethacrylate), blended with TEGDMA (triethylene glycol dimethacrylate) as a diluent. TEGDMA has a lower molecular weight than Bis-GMA and due to higher number of double bonds per unit, more covalent cross-links are created during polymerization, resulting in a relatively higher shrinkage rate.^10^ Polymerization shrinkage and subsequent dimensional change can cause internal stress at tooth‒adhesive interface, which in turn causes debonding, microleakage and secondary caries or enamel fractures.^22^ Accordingly, some manufacturers have replaced the majority of TEGDMA with UDMA (urethane dimethacrylate) and Bis-EMA (bisphenol-A polyethyleneglycol diether dimethacrylate), which results in less shrinkage and less moisture sensitivity.^10^

Moreover, incorporation of fibers into dental polymers has resulted in an improvement in mechanical characteristics. They are capable of resisting tensile stress and may act as a crack stopper. They enhance fracture resistance by either increasing crack blunting or providing sites for energy dissipation during crack propagation through delamination.^9^ According to the manufacturers' information in Table 1, Z100, Z250 and Nulite F are various composite resins with different filler sizes and contents and dissimilar matrix compositions. Nulite F is a Bis-GMA hybrid composite resin reinforced with glass micro-rods to produce a composite resin with exceptionally high strength and extraordinary fracture resistance. On the other hand, Z100 consists of Bis-GMA and TEGDMA while in Z250 most of TEGDMA has been replaced with a mixture of UDMA and Bis-EMA. The filler contents of Z100 and Z250 are similar and consist of zirconium and silica particles ranging from 0.01 to 3.5 μm and an average particle size of 0.6 μm loaded to 66% and 60% by volume, respectively.^23^ Although filler load by volume in Z100 is more than that in Z250, the latter contains more small particles than does Z100.^24^

According to Bailey and Swift, bleaching procedures affect composite resins due to their concentration of organic matrix.^5^ Therefore, this finding could explain how the matrix resin concentrations have affected microleakage of these three composite resins after bleaching, since Nulite F has the least matrix concentration and Z250 has the most.

Bailey and Swift also observed cracks between the resin matrix and particles, in SEM analysis.^5^ These interactions in enamel and composite resin subsequent to bleaching treatment may alter the coefficient of thermal expansion in the enamel and restorative materials, possibly leading to marginal leakage.^25^ However, in Nulite F fiber incorporation helps stop crack propagation.

In the present study, higher concentration of the low molecular-weight monomers with more degree of conversion might also explain the resistance of Nulite F to destructive effects of carbamide peroxide, which provides excellent results after bleaching procedures. On the other hand, higher molecular-weight monomers in Z250 result in lower degree of conversion and more unreacted monomers in the matrix seem to be more adversely affected by bleaching agents.

In the current study, in control groups, there was a statistically significant difference in gingival microleakage. G3 showed the lowest and G5 demonstrated the highest microleakage scores. Although some studies have shown that in regular or packable composite resins, contraction stress is directly proportional to filler content, regardless of differences in matrix composition,^11^ Lee and Park reported that the space occupied by the filler particles does not have a role in polymerization shrinkage. They claimed that high filler loads require low molecular-weight monomers to ensure proper handling viscosity; therefore, within certain limits, polymerization shrinkage does not depend on filler load. The lower molecular-weight monomer, added to control the handling viscosity in packable composite resins, may be responsible for higher shrinkage values.^26^ Hence, reducing the TEGDMA content and replacing it with larger monomers such as UDMA, which has a higher molecular weight, can increase viscosity and reduce polymerization shrinkage.^10,27^ Therefore, as Z100 and Nulite F matrix base is Bis-GMA, despite their higher filler load, it is rational to believe that their polymerization shrinkage and consequently their microleakage might be higher than those of Z250. Palin et al and Fleming et al reported that polymerization shrinkage in Z100 was more than that in Z250,^23,28^ consistent with other studies.^29-32^ Chung et al in an unpublished research showed that the mean percentage of shrinkage, 120 seconds after polymerization, was maximum in Nulite F and minimum in Z250.^33^ Based on the results of the present study, the gingival microleakage in G5 was higher than G1 and the latter was higher than G3. Therefore, it might be concluded that the polymerization shrinkage was capable of forming gaps, resulting in subsequent microleakage. Neiva et al have also suggested that the polymerization shrinkage may be one of the main factors directly responsible for microleakage^34^ and Calheiros et al verified this idea in their study and proved a direct relationship between polymerization shrinkage and microleakage.^35^ However, this outcome was not confirmed by Palin et al and Fleming et al.^23,28^

In this study, the control groups did not show a statistically significant difference in incisal margin microleakage. This resulted from a more durable bond to incisal enamel that resisted polymerization shrinkage stress. Mortazavi et al also concluded that bleaching did not affect the incisal microleakage of Z250 composite restorations.^36^

The bonding agents that were used in this study were both total-etch and two-step adhesives. Bonding agents and composite resins, which were utilized together, were chosen from the same manufacturer. Although the bonding agents were similar in G1 and G3, there was higher gingival microleakage in G1. Therefore, contrary to a report by Chimello et al, who described using the same bonding agent resulting in the same microleakage,^2^ we could deduce that bonding agents had no influence on microleakage, consistent with the results reported by Sharaffedin and Varachehre, who concluded that there was no difference in microleakage between different bonding agents used with the same composite resin.^37^ Therefore, differences in sealing abilities of these three composite resins at gingival margins can be justified by the different shrinkage rates, primarily depending on the composite resin matrix composition, rather than filler type, size and load or the bonding agent used. The matrixes, which had greater amounts of high molecular-weight monomers, such as UDMA and Bis-EMA, exhibited less polymerization shrinkage and less microleakage. Therefore, the null hypothesis of this study was not refuted completely.

Finally, due to lack of long-term in vivo studies to confirm these reports, it is inevitably necessary to periodically follow patients who undergo any type of bleaching treatments. The patients should also be informed that bleaching might adversely affect their composite resin restorations. Further studies with various types of materials are recommended.

## Conclusion

Based on the results and limitations of this in vitro study, it seems that gingival microleakage of bleached composite resins were related to their matrix composition and filler type rather than the filler load. Bleaching had the least negative effect on fiber-reinforced composite resin (Nulite F) and the most adverse effect was seen on particle-filled composite resin (Z250). In unbleached composite resins, microleakage was the outcome of polymerization shrinkage and the role of matrix composition was more obvious than filler type, size and load.

##  Acknowledgments

This article was prepared based on a doctoral thesis and a research project (#1186) approved by the Medical Ethics and Research Office at Shiraz University of Medical Sciences.
